# Application of Real-time Ultrasound Elastography in Diagnosing Benign and Malignant Thyroid Solid Nodules

**DOI:** 10.3969/j.issn.2095-3941.2012.02.008

**Published:** 2012-06

**Authors:** Hai-ling Wang, Sheng Zhang, Xiao-jie Xin, Li-hui Zhao, Chun-xiang Li, Jia-li Mu, Xue-qing Wei

**Affiliations:** Department of Ultrasonographic Diagnosis and Therapy, Tianjin Medical University Cancer Institute and Hospital, Tianjin 300060, China

**Keywords:** ultrasound elastography, elasticity scores, strain ratio, thyroid solid nodule

## Abstract

**Objective:**

Real-time ultrasound elastography (US-E) is a helpful tool in diagnosing thyroid nodules. This study aims to evaluate thyroid solid nodules, to establish the accuracy of US-E in providing information on the nature of these nodules, and to assess the clinical value of elasticity scores (ES) and strain ratio (SR) in differentiating thyroid solid nodules and to explore its distribution characteristics using pathological analysis as reference.

**Methods:**

Traditional ultrasonography and US-E were performed on 131 thyroid solid nodules (99 benign ones and 32 malignant ones) in 120 patients (78 females and 41 males). Three radiologists evaluated the nodules based on a four-degree elasticity scoring system. The nodules were classiﬁed according to the ES as soft (ES 1-2) or hard (ES 3-4). The SR was calculated online.

**Results:**

The sensitivity and speciﬁcity of the ES for thyroid cancer diagnosis were 78% and 80%, respectively. SR values ≥ 2.9 used as a standard to distinguish benign from malignant nodules had a sensitivity of 87% and a specificity of 92%. The SR of the benign lesions was 1.64±1.37, which was significantly different from that of malignant lesions, which was 4.96±2.13 (*P*<0.01).

**Conclusions:**

Both the ES and SR were higher in malignant nodules than those in benign ones. Real-time US-E was a useful index in the differential diagnosis of thyroid solid nodules. It can provide quantitative information on thyroid nodule characterization and improve diagnostic confidence.

## Introduction

The nodular thyroid disease affects the general population, particularly among iodine-deficient individuals. Thyroid nodules are observed in 5% of the subjects ^[^^1^^, ^^2^^]^, but are detected in about 50% of the general population via thyroid ultrasound ^[^^3^^-^^5^^]^.

The basic principle of ultrasound elastography (US-E) is that the compression of the examined tissue produces a strain, which is smaller in hard tissues than in soft tissues. The results of this technique are scored by measuring the degree of distortion of the ultrasound beam while an external force is applied ^[^^6^^]^. Malignant lesions are often characterized by greater stiffness than in normal tissues ^[^^7^^]^. The aim of this study is to evaluate the clinical value of the elasticity score via US-E in the differential diagnosis of thyroid solid nodules.

## Patients and Methods

### Patients

This study was approved by the Tianjin Medical University Cancer Institute and Hospital. A total of 131 nodules in 120 patients (78 females; mean age, 44.31 years; range, 18 to 70 years; and 41 males; mean age, 49.92 years; range, 24 to 69 years) were selected from January to November 2011. The inclusion criterion was the presence of solid lesions in one thyroid lobe. The mean size of the nodules was 1.91 cm (within a range of 0.87 to 3.32 cm). Surgical results were used as the reference standards.

### Imaging acquisition

The patients were in a supine position with the neck slightly extended. A considerable amount of ultrasound gel was applied to the patients’ neck as a standoff pad. Both conventional sonography and real-time US-E were performed using a Philips IU22 system equipped with a liner probe with a central frequency of 5 MHz to 12 MHz. All examinations were conducted and recorded by three experienced sonographers. Two of these sonographers had more than eight years of experience in sonography and about two months of special training in elastography.

Based on the preliminary experiments, the probe should slightly come into contact with the skin because a strong initial compression may increase the probability of false negatives. A region of interest (ROI) was centered on the lesion, including sufﬁcient surrounding thyroid tissue. The great cervical vessels were avoided as much as possible.

### Elastogram review

#### Evaluation based on elasticity scores

**Figure 1:**
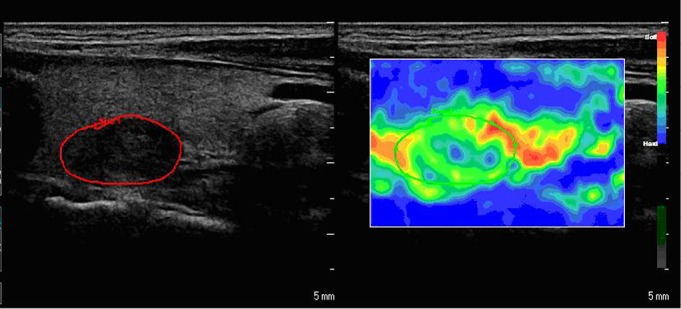
A nodular goiter with a mean elasticity score of 1 in a 41-year-old woman. The average strain ratio was 1.20.

**Figure 2:**
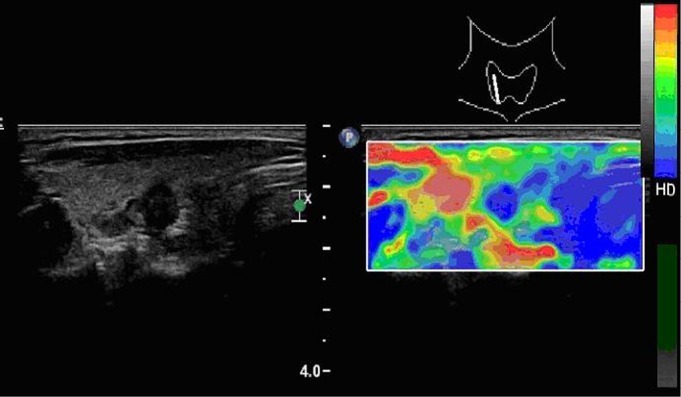
A papillary carcinoma (follicular variant) with elasticity score of 4 in a 56-year-old woman. The average strain ratio of the nodule was 5.23.

Each nodule was assigned an elasticity score based on the pattern type according to the classiﬁcation proposed by Fukunari ^[^^8^^]^. Pattern 1 (score 1): most of the nodule is displayed in green. Pattern 2 (score 2): the center of the nodule is displayed in green and its peripheral part in blue. Pattern 3 (score 3): the nodule is displayed as a mixture of red, green, and blue. Pattern 4 (score 4): the entire nodule is displayed in blue (**Figures 1**** and ****2**).

#### Evaluation based on strain ratio

The sonographers performed this evaluation during the examination using a software that was connected to the machine. The best-ﬁt 2D sonogram-elastogram image pairs were selected, and strain ratio (SR) was evaluated. The operators were first asked to trace area A manually along the borderline of the lesion. Area B was then selected just beside the target lesion as reference. The homogeneous thyroid tissue at the same depth was chosen because the strain varies as a function of depth. The lesions were used as the reference tissue. The software could automatically calculate for the SR. Each lesion was assessed at least three times based on different static images, and the average value was recorded as the ﬁnal result. The radiologists performed the examination and evaluation for about 5 to 8 min per patient.

## Results

### Pathologic findings

All 131 nodules with a final postoperative histological diagnosis were studied. Among the 32 malignant nodules, 27 were papillary thyroid carcinomas (13 classic variant cases, 7 follicular variant cases, 3 tall cell variant cases, and 2 trabecular variant cases), 3 were follicular carcinomas, 1 was adenoma with foci (3 mm) of papillary carcinoma, and 1 was an undifferentiated carcinoma. The remaining 99 nodules were benign based on histological results. Of these, 77 were nodular goiters and 22 were adenomas.

### US elastography

#### Elasticity scores of 131 thyroid nodules

Eighty-nine of the 131 nodules had scores of 1 or 2, and 82 of these nodules were diagnosed as benign. Forty-two of the 131 nodules had a score of 3 or 4, and 25 of these nodules were diagnosed as malignant. Thus, scores 3 and 4 were indicative of malignancy, with a sensitivity of 78% and a speciﬁcity of 80% (**Table 1**). All nodules without calciﬁcation and had an elasticity score of 1 or 2 were considered benign (**Figure 1**).

**Table 1:** Association between elastographic pattern and malignancy in the nodules.

	Benign nodules, *n* (%)	Malignant nodules, *n* (%)	Total nodules, *n* (%)
Score 1 and 2	82 (63)	7 (5)	89 (68)
Score 3 and 4	17 (13)	25 (19)	42 (32)
Total	99 (76)	32 (24)	131

#### Elastography ratio of 131 thyroid nodules

**Figure 3:**
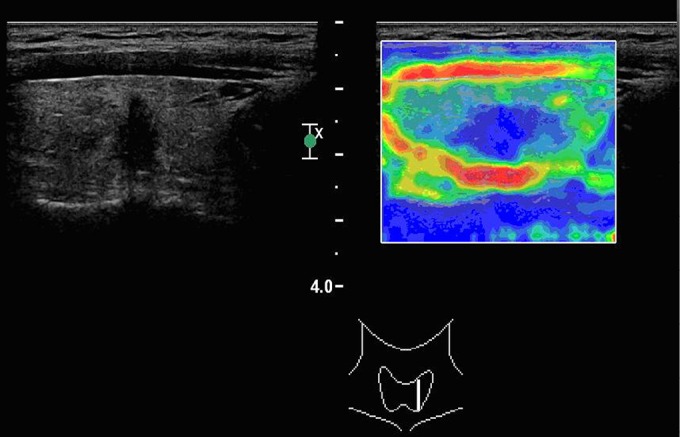
A papillary carcinoma (classic variant) with elasticity score of 4 in a 38-year-old man. The average strain ratio of the nodule was 4.98.

Ninety-six of the 131 nodules had SR<2.9, and 92 of these nodules were benign. Thirty-five of the 131 nodules had SR≥2.9 and 28 were malignant (**Table 2**). This criterion had a sensitivity of 87% and a speciﬁcity of 92% (**Figures 2**** and ****3**).

**Table 2:** Association between strain ratio and malignancy in the nodules.

	Benign nodules, *n* (%)	Malignant nodules, *n* (%)	Total nodules, *n* (%)
Strain ration﹤2.9	92 (70)	4 (3)	96 (73)
Strain ration≥2.9	7 (6)	28 (21)	35 (27)
Total	99 (76)	32 (24)	131

#### Distribution of elastography patterns and SR of the nodules

The elastography patterns and SR of different pathological nodules were different (**Tables 1**** and ****2**). The mean SR for the benign nodules and malignant ones was signiﬁcantly different (1.64±1.37 *vs.* 4.96±2.13, *P*<0.001). In terms of pathological type, the SR was 1.53±1.23 for nodular goiters, 1.76±1.25 for thyroid adenomas, 5.02±2.07 for papillary carcinomas, 4.95±2.12 for follicular carcinomas, and 6.54±0.55 for undifferentiated carcinomas.

## Discussion

Real-time US-E is a newly developed diagnostic tool that evaluates the degree of distortion of ultrasound beam while an external force is applied. It is based on the principle that the softer parts of the tissues deform more easily than the harder parts under compression. Thus, a semi-quantitative determination of tissue elasticity is observed ^[^^9^^]^. Thus far, elastography has been evaluated in two ways, namely, based on the elasticity score and on the strain ratio. Malignant thyroid nodules such as papillary thyroid carcinoma, which is the most common histotype, displayed lower elasticity compared with benign lesions ^[^^10^^-^^14^^]^.

The elasticity score evaluation was based on the color distribution, which was superimposed on the B-mode image. Previous reports found this method to be efficient ^[^^15^^,^^16^^]^. However, information about the stiffness of the target mass is limited. In the present study, the benign and malignant lesions of the elasticity score had a greater extent of overlap, and the sensitivity and specificity were lower.

On the contrary, the SR is a quantitative index that can provide accurate information. In 2007, Koji Waki and Takeshi Matsumura conducted a research using a quantitative phantom and an automatic compressor ^[^^17^^]^. Their findings demonstrated that regardless of stress, the strain ratio showed constant properties, and the value continued to increase with the elasticity ratio. However, the selection of the reference tissue might affect the results. Zhi et al.^[^^18^^]^ recommended the breast tissue at the same depth with the lesions to be used as the reference. Rago et al.^[^^15^^]^ indicated that the predictability of elastography was independent of the nodular size and position. In the present study, the thyroid tissue in the same depth was chosen, and the target nodule was used as the reference. The results revealed that both the score and the SR were repeatable and reliable. The longitudinal view of the thyroid was recommended because it could provide enough reference tissues at the same depth with the lesions.

The distribution of the SR conﬁrmed that the benign nodules were much softer than the malignant ones. In this study, most of the papillary carcinomas were imaged as blue in the elastograms. The value of the SR was also quite high. Compared with papillary carcinoma, follicular and medullary thyroid carcinoma had less ﬁber content and more cellular components. Papillary carcinoma is often accompanied by the formation of sand, which makes the collection of pathologic specimens of papillary carcinoma relatively hard ^[^^19^^]^. Both the ES and SR were higher in malignant nodules than those in benign ones. This result indicated that both the ES and SR are useful indexes in the differential diagnosis of solid thyroid nodules. This method can provide quantitative information on thyroid nodule characterization and improve diagnostic confidence.

Real-time US-E is inﬂuenced by the volume, topography, and number of thyroid nodules. For nodules with diameters larger than 3 cm, elastography might not be performed with the external compression of the whole nodule ^[^^20^^]^. No information about the lower diameter of the thyroid nodules that can be evaluated by real-time US-E is available as of this writing. The approach could only perform a limited evaluation of multi-nodular goiters because the nodules must be clearly distinguishable from one another. The accuracy of real-time US-E might also be altered if the nodules are close to the carotid artery because arterial pulsation may create elastographic images, which affects the ability to acquire adequate data and to interpret them accurately ^[^^20^^]^.

In the present study, many nodules were selected based on suspicious features of malignancy. Thus, the prevalence of the disease within the entire category of nodules with non-diagnostic histology was overestimated.

Upon further investigation, the implications of the findings in the present study on the clinical management of indeterminate or non-diagnostic lesions are likely to be relevant because US-E might restrict the indications of surgical therapy to the subgroup of patients with a higher risk of thyroid cancer. The low number of false negative results using US-E together with the low progression rate of differentiated thyroid cancer might allow most patients to be recommended for follow-up sessions without significant costs in terms of prognosis. US-E could also be helpful in defining the extent of thyroid ectomy (total or lobectomy) for these lesions.

Subtype of thyroid carcinoma: in the meta-analysis of Bojunga et al. ^[^^21^^]^, false negative results were obtained in 10/135 (7%) of the papillary carcinomas and 4/9 (44%) of follicular carcinomas. Correctly diagnosing this subtype of carcinoma might be difficult even at the histopathology level. Their gross anatomy and cellular patterns overlap with those of benign nodules. The sonographic differences between follicular adenomas and carcinomas have not been fully established ^[^^22^^]^. In a retrospective study, Seo et al. ^[^^23^^]^ reported that iso-hypoechogenicity and microcalciﬁcations or rim calciﬁcations were more common in follicular carcinomas than in follicular adenomas. However, in one prospective study, the sensitivity of the ultrasound characteristics was 86.5% for non-follicular neoplasm and 18.2% for follicular carcinomas^[^^24^^]^. In the present study, the mean SR for follicular carcinomas was 4.95±2.12. Compared with other types of cancer, this value is not very different, and indicates that the SR could be helpful in the diagnosis of follicular carcinoma. Further studies involving more cases of follicular adenomas and carcinomas are needed to investigate the role of elastography in their evaluation. In the present study, the elasticity strain ratio of one case of undifferentiated thyroid carcinoma was much higher than the other types of thyroid carcinoma, suggesting that it is the hardest among all pathological types.

In conclusion, real-time US-E is proposed as an important tool in the pre-surgical risk stratification of thyroid cancer in nodules with indeterminate or non-diagnostic cytology, high elasticity, which is highly associated with benign histology, and low elasticity, which is highly associated with malignant histology.
